# Evaluating the Prodromal Questionnaire-16 for Screening and Prediction of Psychosis in Autistic Adults: Cross-Sectional and Longitudinal Findings From a National Cohort

**DOI:** 10.1093/schbul/sbag160

**Published:** 2026-09-16

**Authors:** Djordje Basic, Sander Begeer, Tim Ziermans

**Affiliations:** Department of Clinical, Neuro- and Developmental Psychology, Vrije Universiteit Amsterdam, Van der Boechorststraat 7, 1081 BT Amsterdam, The Netherlands; Department of Clinical, Neuro- and Developmental Psychology, Vrije Universiteit Amsterdam, Van der Boechorststraat 7, 1081 BT Amsterdam, The Netherlands; Department of Psychology, University of Amsterdam, Nieuwe Achtergracht 129-B, 1018 WS Amsterdam, The Netherlands

**Keywords:** autistic traits, attenuated psychotic symptoms, PQ-16, screening

## Abstract

**Background and Hypothesis:**

Autistic individuals are at elevated risk for psychosis, yet attenuated psychotic symptoms may go undetected due to overlapping features and limited validation of screening tools in autistic populations. This study evaluated the performance of the Prodromal Questionnaire-16 (PQ-16) in autistic adults, including its longitudinal predictive value for schizophrenia-spectrum diagnoses, and examined demographic and clinical correlates of attenuated psychotic symptoms.

**Study Design:**

Using data from a large longitudinal cohort (the Netherlands Autism Register), we analyzed self-reported PQ-16 symptom and distress scores alongside demographic variables, age at autism diagnosis, autistic traits (AQ-28), and depressive symptoms (PHQ-Dep-4). Descriptive analyses characterized PQ-16 score distributions. Linear regression models examined predictors of PQ-16 symptom severity. Logistic regression and ROC analyses evaluated whether PQ-16 scores predicted self-reported new-onset schizophrenia-spectrum diagnoses at follow-up.

**Study Results:**

Among 1686 autistic adults (mean age = 46.04), 34.1% exceeded the PQ-16 cutoff (≥6), indicating a substantially higher prevalence of attenuated psychotic symptoms than reported in non-autistic populations. In the fully adjusted model, higher PQ-16 scores were independently associated with greater autistic trait severity and depressive symptoms. New-onset schizophrenia-spectrum diagnoses based on self-report occurred in 1.4% of participants with longitudinal data (5/361) and were observed exclusively among individuals above the PQ-16 cutoff (3.2% vs 0.0%, *P* = .015). ROC analyses suggested apparent discrimination for PQ-16 total, distress, and combined scores, although estimates should be interpreted cautiously given the very small number of incident cases.

**Conclusions:**

The PQ-16 identifies meaningful patterns of attenuated psychotic symptoms in autistic adults. Findings support brief screening as part of a 2-step assessment approach.

## Introduction

Autistic individuals are at elevated risk for developing schizophrenia-spectrum disorders (SSDs), with converging evidence from clinical, epidemiological, and neurodevelopmental research. Meta-analyses estimate that 4%-9% of autistic adults meet criteria for SSDs.^1–3^ These figures are substantially higher than those in the general population (approximately 0.5%).^1^^,^^3^ Register-based studies from the Netherlands and Sweden similarly show that autistic individuals are over 5 times more likely to develop non-affective psychotic or bipolar disorders than non-autistic peers, even after adjusting for confounders.^4^^,^^5^ Yet psychosis is often under-recognized in autism, due to overlapping features, diagnostic overshadowing, and limited screening tools validated for this population.^6^^,^^7^

Theoretical work has challenged traditional diagnostic boundaries, emphasizing shared transdiagnostic features between autism and SSDs. Cognitive and behavioral similarities, such as atypical social cognition, sensory processing difficulties, social withdrawal, and unusual perceptual experiences, are observed in both conditions.^8–10^ Family and twin studies, genome-wide association studies, and neuroimaging research all support shared genetic and neurocognitive mechanisms.^11–16^ Both conditions are highly heritable,^11^^,^^12^ and common genetic variants have been identified across autism and SSD.^13–16^

Building on these shared features, a central question remains: How should the relationship between autism and SSD be conceptualized? Chisholm et al. outlined 8 co-occurrence models, ranging from a shared continuum to entirely distinct yet co-occurring conditions.^7^ Integrative frameworks propose that common neurodevelopmental mechanisms, such as impaired social prediction, executive control, or salience attribution, may lead to divergent outcomes depending on developmental context.^17^ Adding further nuance, recent studies have highlighted disturbances in basic self-experience as features that help differentiate schizophrenia from autism. These include a disrupted sense of agency, bodily disconnection, and altered self-world boundaries, which are more frequently observed in schizophrenia and may precede full-blown symptoms by several years.^18–20^

While these models improve conceptual clarity, they also pose clinical challenges: How can early psychosis be detected in autistic individuals, given overlapping presentations? Research has therefore increasingly focused on attenuated psychotic symptoms and other psychosis-spectrum experiences, sometimes described as part of the clinical high-risk (prodromal) state, as possible transdiagnostic vulnerability markers. These include mild delusional thoughts, perceptual anomalies, or unusual beliefs. In clinical high-risk populations (CHR-Ps), such symptoms predict transition to psychosis in 20%-30% of individuals within 3 years.^21^ Preliminary studies suggest comparable transition rates in autistic youth with similar symptom profiles,^22^^,^^23^ although findings on symptom prevalence are mixed. Some studies report elevated subclinical symptoms in autistic populations,^24^ while others find no group-level increase.^25^ Rather than indicating a stable, trait-like expression of symptoms in autism, these inconsistencies may instead reflect state-like fluctuations over time, consistent with broader findings that attenuated psychotic symptoms are often transitory in nature.^25^

A lack of validated screening tools for autism compounds these diagnostic ambiguities. Standard early detection protocols follow a 2-step approach: first, brief self-report screeners, such as the Prodromal Questionnaire (eg, PQ-B^26^ or PQ-16^27^), are used to detect subthreshold symptoms; second, structured interviews such as the Comprehensive Assessment of At-Risk Mental States (CAARMS)^28^ or the Structured Interview for Prodromal Syndromes (SIPS)^29^ are used to establish CHR-P status. However, these instruments were developed for non-autistic or help-seeking populations and may misclassify core autistic traits, like perceptual sensitivity or unusual thinking, as prodromal indicators. The PQ-16, although efficient and psychometrically sound in general samples,^30^ has not been validated in individuals with autism. To date, only 2 child-focused studies have examined PQ variants in the context of autism.^31^^,^^32^ Our study addresses this gap by evaluating the distribution and predictive value of the PQ-16 in a large cohort of adults with autism. We assess score distribution, demographic and clinical predictors of PQ-16 symptom severity, and whether PQ-16 scores predict new-onset psychosis. Together, these findings provide a clearer understanding of whether the PQ-16 can detect clinically meaningful attenuated psychotic symptoms in autism.

## Methods

### Participants and Procedures

This study utilized data from the Netherlands Autism Register (NAR), an ongoing longitudinal cohort that collects annual self-report survey data from autistic individuals and their families in the Netherlands. Participants voluntarily enrolled in the registry and completed online questionnaires assessing demographic, clinical, and psychosocial domains.

The present study used data from wave 8 (2022) and wave 9 (2023), as the PQ-16 was not available in earlier waves. Participants were eligible for inclusion if they (1) were aged ≥16 years, (2) had a formal autism diagnosis confirmed by an independent clinician, and (3) completed the PQ-16 in wave 8 or wave 9. The final analytic sample included 1686 individuals aged 16-88 years (M = 46.04, SD = 14.01). Although the sample included both youth (16-18 years) and adults, the majority of participants were adults, and the sample is therefore referred to as adults throughout.

We conducted both cross-sectional and longitudinal analyses. Cross-sectional models used data from the most recent wave available per participant. Longitudinal prediction models included only those with valid data in both wave 8 and wave 9. Participants with a prior self-reported psychosis diagnosis before baseline were excluded from the longitudinal analyses. Descriptive characteristics of the sample are summarized in Table 1.

**Table 1:** Descriptive Characteristics of the Sample (*N* = 1686).

**Variable**	***N***	**Range/categories**	**Mean (SD)/*N* (%)**
Age (latest)	1683	16.36-87.98	46.04 (14.01)
Age of autism diagnosis	1600	0.67-71.33	36.38 (15.17)
AQ28—Imagination	1652	8-32	22.19 (4.56)
AQ28—Numbers & Patterns	1652	5-20	13.54 (3.73)
AQ28—Routine	1652	4-16	12.36 (2.31)
AQ28—Social Behavior	1652	28-80	60.43 (7.97)
AQ28—Social Skills	1652	7-28	21.51 (3.74)
AQ28—Switching	1652	5-16	13.04 (2.15)
AQ28—Total Score	1652	44-112	82.64 (10.76)
PQ-16 Total Score (latest)	1681	0-16	4.49 (3.26)
PQ-16 Distress Score (latest)	1684	0-40	5.72 (5.71)
PHQ-Dep-4	947	4-16	7.83 (3.00)
Sex (at birth)	1686	Male	620 (36.8%)
		Female	1062 (63%)
		Other^**a**^	4 (0.2%)
Perceived gender^**b**^ (latest)	1686	Man	580 (34.4%)
		Woman	873 (51.8%)
		Other^c^	233 (13.8%)
Education^**d**^ (CBS-coded)	1686	Missing/unknown	390 (23.1%)
		Low	128 (7.6%)
		Middle	356 (21.1%)
		High	812 (48.2%)
Employment status (latest)	1686	Yes	884 (52.4%)
		No	802 (47.6%)
Schizophrenia-spectrum comorbidity^e^	1686	Yes	18 (1.1%)
		No	1668 (98.9%)

All percentages are valid percentages. Abbreviations: AQ-28 = Autism Spectrum Quotient Short Form; PQ-16 = The Prodromal Questionnaire; PHQ-Dep-4 = Patient Health Questionnaire (4 depression items).

a Gender could not be determined (*N* = 2), and I’d rather not say that (*N* = 2) combined.

b Responses at wave 8 (*N* = 243) and wave 9 (*N* = 1443) combined.

c Included non-binary, neutral, unknown, or self-described identities.

d Education was categorized according to the CBS (Statistics Netherlands) classification system, using 3 levels: Low, Middle, and High.

e Schizophrenia-spectrum comorbidity reflects the presence of a self-reported diagnosis of schizophrenia or other psychotic disorder based on the most recent available data (wave 9 if available, otherwise wave 8).

### Ethical Considerations

This study was approved by the Scientific and Ethical Review Board of the Faculty of Behavioural and Movement Sciences at Vrije Universiteit Amsterdam (VCWE-2023-024R1). All participants provided informed consent for the use of anonymized data for scientific research. Study procedures were conducted in accordance with the principles outlined in the Declaration of Helsinki. Study design, hypotheses, and primary analyses were preregistered on the Open Science Framework (OSF; DOI: 10.17605/OSF.IO/H9QFW). Additional information on the NAR is described in detail in the cohort protocol paper by Jonkman et al.^33^ and available at https://nar.vu.nl.

## Measures

### Psychosis-Like Experiences (Prodromal Questionnaire, 16-Item Version)

The Prodromal Questionnaire, 16-item version (PQ-16)^27^ is a self-report screener designed to assess attenuated psychotic symptoms. Each item is rated as “True” (1) or “False” (0) and, if endorsed, rated for associated distress on a 4-point scale from 0 (“No distress”) to 3 (“Severe distress”). The total PQ-16 Symptom Score is calculated as the sum of all “True” responses (range: 0-16) and the PQ-16 Distress Score as the sum of distress ratings across endorsed items (range: 0-48).

The Dutch translation of the PQ-16 has demonstrated good internal consistency (Cronbach’s α = .77) and strong diagnostic accuracy in general help-seeking populations, with sensitivity and specificity of 87% at a cutoff of ≥6 for predicting clinical high risk (CHR) status based on the CAARMS interview, rather than psychosis diagnoses per se.^27^ The most recent systematic review further supports its utility across diverse populations while highlighting potential concerns about false positives in groups such as autistic individuals, where overlapping sensory or cognitive features may inflate scores despite the absence of psychotic risk.^30^ In the current study, the total symptom score (range: 0-16) was used as the primary outcome, with a validated cutoff score of ≥6 to identify individuals with attenuated psychotic symptoms.

### Autistic Traits (28-item Autism Spectrum Quotient Short Form)

Autistic traits were assessed using the 28-item Autism Spectrum Quotient Short Form (AQ-28),^34^ a self-report measure that evaluates core autism characteristics across social and non-social domains. The AQ-28 includes subscales assessing 6 trait domains: Social Skills, Social Behavior, Switching, Routine, Imagination, and Numbers & Patterns. Items were rated on a 4-point Likert scale (1 = *definitely agree* to 4 = *definitely disagree*), with reverse scoring for items in which an “agree” response is characteristic of autism. Total scores were calculated using the full Likert scoring (range: 28-112) with higher scores indicating more pronounced autistic traits. The AQ-28 demonstrates good internal consistency (Cronbach’s α ≈ .85) and strong convergence with the full 50-item version while reducing participant burden.^34^

### Depressive Symptoms (4-item Patient Health Questionnaire)

Depressive symptoms were measured using the 4-item Patient Health Questionnaire (PHQ-Dep-4),^35^ a brief screening tool assessing core depressive symptoms over the past 2 weeks. Items are rated on a 4-point Likert scale (1 = *not at all* to 4 = *nearly every day*), with higher scores indicating more depressive symptoms (range: 4-16). Internal consistency in the present sample was good (Cronbach’s α = .79). Prior research supports the reliability and validity of PHQ-based depression measures across diverse populations, with evidence suggesting comparable performance of brief PHQ variants relative to longer forms such as the PHQ-8 and PHQ-9.^36^

### Sociodemographic and Clinical Variables

Age was treated as a continuous variable based on the most recent available self-report, using wave 9 (2023) data when available and, otherwise, wave 8 (2022). Perceived gender was harmonized by using wave 9 responses when available and wave 8 otherwise. Gender identity was then categorized as man, woman, or other (including non-binary, neutral, unknown, or self-described identities). Sex assigned at birth was coded as a binary categorical variable (male, female), with a third category “other” for responses such as “sex could not be determined” or “I’d rather not say.” Employment status was assessed with the question “Are you currently employed (with an employer or as a self-employed person)?” at both wave 8 and wave 9; the most recent response was used to construct a binary employment variable.

Antipsychotic and antidepressant medication use was reported at both wave 8 and wave 9. For descriptive analyses, the most recent available response was used (wave 9; if available, otherwise, wave 8). Schizophrenia-spectrum comorbidity was assessed using self-reported diagnoses of schizophrenia or other psychotic disorders at wave 8 and wave 9; no independent clinical validation of these reports is available within the NAR. For longitudinal analyses, new-onset psychosis was defined as a new diagnosis reported at wave 9 among those without a diagnosis at wave 8 and with data at both waves.

### Statistical Analyses

All analyses were conducted using IBM SPSS Statistics version 29.0.1.0.^37^ Descriptive statistics were reported with means and standard deviations for continuous variables (eg, age, age at diagnosis, AQ-28, PQ-16, PHQ-Dep-4) and frequencies and valid percentages for categorical variables (eg, sex, perceived gender, education, employment, schizophrenia-spectrum comorbidity, binary PQ-16 scores).

First, we examined the distribution of PQ-16 symptom scores using histograms and summary statistics. Item-level endorsement frequencies were calculated for each PQ-16 item and stratified into 3 symptom severity groups: low (0-2 symptoms), medium (3-5 symptoms), and high (≥6 symptoms). These patterns are reported in Supplementary Table S1 and Figure S1. Next, multiple linear regression models were fitted to examine whether age, sex, autistic traits (AQ-28), and depressive symptoms (PHQ-Dep-4) predicted PQ-16 symptom scores. All continuous predictors were standardized to z-scores. Binary logistic regression was used to test whether PQ-16 symptom and distress scores at wave 8 predicted new-onset schizophrenia-spectrum diagnoses at wave 9. Receiver operating characteristic (ROC) analyses and diagnostic accuracy indices were calculated for selected cutoffs (eg, PQ-16 ≥ 6, ≥7, ≥8, ≥9). In preregistered exploratory analyses, we also tested for interaction effects (eg, AQ × Gender) and conducted sensitivity analyses including log-transformed symptom scores and AQ tertile splits. To examine the potential impact of age on predictive analyses, a post hoc sensitivity analysis was conducted in a subsample restricted to participants aged ≤40 years at baseline, reflecting the typical high-risk period for psychosis onset.

Additional exploratory analyses were conducted to examine whether outcomes differed by age at the time of autism diagnosis. A binary variable was created using a 12-year cutoff to distinguish childhood-diagnosed (≤12 years) from later-diagnosed (>12 years) participants. This cutoff was selected post hoc as a pragmatic threshold. Age 12 is commonly used as a boundary between childhood and adolescence, and a diagnosis before this age is more likely to reflect a “classic” early developmental presentation identified during primary school years. Group differences were assessed using Fisher’s exact tests (for new-onset psychosis) and chi-square tests (for elevated PQ-16 scores). Given the exploratory nature of these analyses, alternative age cutoffs were not examined to avoid instability in the models.

Model assumptions were checked using residual plots, variance inflation factors (VIFs), and diagnostic statistics. All *P*-values were reported to 3 decimal places. Effect sizes (eg, standardized β coefficients, odds ratios) and 95% confidence intervals were reported for all regression models. Multiple comparisons were adjusted using the Benjamini–Hochberg procedure.^38^

Due to high completion rates in the NAR, item-level missingness was minimal. No imputation procedures were applied; analyses were conducted using available cases, with valid sample sizes reported per model.

## Results

### Sample Characteristics

The analytic sample included 1686 autistic youth and adults (*M* = 46.04, *SD* = 14.01; range = 16-88). On average, participants received their autism diagnosis in adulthood (*M* = 36.38, *SD* = 15.17, *median* = 37.71). Regarding sex assigned at birth, 63% of participants were female, and 36.8% were male (0.2% other). Regarding current gender identity, 51.8% identified as women, 34.4% as men, and 13.8% as outside the gender binary. Educational attainment was relatively high (48.2% with higher education), yet only 52.4% were employed. 12.5% of participants reported antipsychotic use and 23.5% reported antidepressant use at wave 8, with similar proportions observed at wave 9 (11.8% and 21.3%, respectively). Schizophrenia-spectrum comorbidity was rare overall (*n* = 18/1686; 1.1%), with new-onset cases emerging in 1.4% of the longitudinal subsample (*n* = 5/361). Full descriptive characteristics are presented in Table 1.

### Prevalence & Correlates of PQ-16 Symptom Severity

Across the sample, the mean PQ-16 total score was 4.49 (*SD* = 3.26; *median* = 4.00, *IQR* = 5.00), and the mean distress score was 5.72 (*SD* = 5.71; *median* = 4.00, *IQR* = 7.00). Using the most recent available data (wave 9; if available, otherwise, wave 8), 34.1% of participants (*n* = 573; 95% *CI* [31.8%, 36.4%]) scored above the established PQ-16 cutoff (≥6), indicating attenuated psychotic symptoms according to the screener. Individuals above the PQ-16 cutoff were slightly younger (*M* = 44.34 vs 46.90 years, *P* < .001), diagnosed with autism at an earlier age (*M* = 34.96 vs 37.03, *P* = .010), and exhibited significantly higher AQ-28 scores, particularly in cognitive rigidity (eg, Numbers & Patterns, *P* < .001; Switching, *P* < .001) and social difficulties (ie, Social Skills, *P* = .004). They also reported significantly higher depressive symptoms on the PHQ-Dep-4 (*M* = 9.10 vs 7.28, *P* < .001). Greater gender diversity was observed in the elevated symptom group (17.8% vs 11.7%, *P* = .002), along with lower employment rates (47.8% vs 55.1%, *P* = .013). Medication use was higher in the elevated PQ-16 group for antipsychotics (14.0% vs 10.6%, *P* = .046), benzodiazepines (14.3% vs 10.2%, *P* = .013), and stimulants (4.7% vs 2.4%, *P* = .012) but did not differ for antidepressants (21.1% vs 21.4%, *P* = .897). Confirmed new-onset psychosis-spectrum comorbidity between waves was significantly more frequent (3.2% vs 0.0%, *P* = .015) in the elevated PQ-16 symptom group. Complete stratified comparisons are presented in Table 2.

**Table 2:** Descriptive Characteristics of Autistic Adults With and Without Elevated Prodromal Psychosis Symptoms (PQ-16 ≥ 6).

**Variable**	**Without elevated psychosis symptoms** ^**a**^ **(*N* = 1108; 65.9%)**	**Elevated psychosis symptoms** ^**b**^ **(*N* = 573; 34.1%)**	**Test statistic** ^**c**^	**df/U**	***P***	**95% CI**
Age (latest)	*N* = 1106; M = 46.90 (SD = 13.92)	*N* = 572; M = 44.34 (SD = 14.08)	3.55	1676	<.001	2.55 [1.14, 3.97]
Age of diagnosis	*N* = 1055; M = 37.03 (SD = 15.21)	*N* = 540; M = 34.96 (SD = 15.06)	2.58	1593	.010	2.07 [0.49, 3.64]
AQ28—Imagination	*N* = 1086; M = 22.33 (4.44)	*N* = 561; M = 21.89 (4.76)	1.86	1645	.063	0.44 [−0.02, 0.91]
AQ28—Numbers & Patterns	*N* = 1086; M = 13.06 (3.76)	*N* = 561; M = 14.48 (3.48)	–7.48	1645	<.001	–1.43 [–1.80, –1.05]
AQ28—Routine	*N* = 1086; M = 12.21 (SD = 2.34)	*N* = 561; M = 12.65 (SD = 2.23)	–3.63	1645	<.001	–0.44 [–0.67, –0.20]
AQ28—Social Behavior	*N* = 1086; M = 60.16 (SD = 7.81)	*N* = 561; M = 60.90 (SD = 8.26)	–1.77	1645	.077	–0.73 [–1.55, 0.08]
AQ28—Social Skills	*N* = 1086; M = 21.31 (SD = 3.70)	*N* = 561; M = 21.86 (SD = 3.77)	–2.86	1645	.004	–0.56 [–0.94, –0.18]
AQ28—Switching	*N* = 1086; M = 12.89 (SD = 2.15)	*N* = 561; M = 13.32 (SD = 2.12)	–3.91	1645	<.001	–0.44 [–0.65, –0.22]
AQ28—Total Score	*N* = 1086; M = 81.80 (SD = 10.59)	*N* = 561; M = 84.21 (SD = 10.92)	–4.33	1645	<.001	–2.41 [–3.50, –1.32]
PHQ-Dep-4	*N* = 655; M = 7.28 (SD = 2.74)	*N* = 280; M = 9.10 (SD = 3.19)	−8.325	461.27	<.001	−1.816 [−2.24, −1.39]
Sex (at birth)	Male 404 (36.5%)	Male 214 (37.3%)	χ^2^ = 1.58	2	.455	—
	Female 703 (63.4%)	Female 375 (62.3%)				
	Other 1 (0.1%)	Other 2 (0.3%)				
Perceived gender	Man: 383 (34.6%)	Man: 195 (34.0%)	χ^2^ = 12.34	2	.002	Greater gender diversity in elevated symptoms group
	Woman: 595 (53.7%)	Woman: 276 (48.2%)				
	Other: 130 (11.7%)	Other: 102 (17.8%)				
Education (CBS-coded)	Low 86 (7.8%)	Low 42 (7.3%)	χ^2^ = 12.10	3	.007	Elevated symptoms group more likely missing/mid-educated
	Middle 217 (19.6%)	Middle 138 (24.1%)				
	High 564 (50.9%)	High 244 (42.6%)				
	Missing 241 (21.8%)	Missing 149 (26.0%)				
Employment status	Yes 489 (55.1%)	Yes 211 (47.8%)	χ^2^ = 6.17	1	.013	Elevated symptoms group less often employed
	No 399 (44.9%)	No 230 (52.2%)				
Medication use (latest)^d^						Antipsychotic, benzodiazepines and use stimulants more common in elevated symptoms group
Antipsychotics	118 (10.6%)	80 (14.0%)	χ^2^ = 3.99	1	.046	—
Antidepressants	237 (21.4%)	121 (21.1%)	χ^2^ = 0.02	1	.897	—
Benzodiazepines	113 (10.2%)	82 (14.3%)	χ^2^ = 6.23	1	.013	—
Stimulants	27 (2.4%)	27 (4.7%)	χ^2^ = 6.29	1	.012	—
Z-drugs	13 (1.2%)	10 (1.7%)	χ^2^ = 0.92	1	.339	—
Mood stabilizers/AEDs	3 (0.3%)	0 (0.0%)	—	—	.555^e^	—
Non-stimulant ADHD meds	1 (0.1%)	1 (0.2%)	—	—	1.000^e^	—
Psychosis (new-onset)	Yes 0 (0.0%)	Yes 5 (3.2%)	—	—	.015^e^	New-onset psychosis more common in elevated symptoms group
	No 203 (100%)	No 153 (96.8%)				

Values reflect valid percentages. Continuous variables were compared using independent-samples *t*-tests when normality assumptions were met; otherwise, Mann–Whitney *U* tests were used. Categorical variables were compared using χ^2^ tests. Fisher’s exact test was used when expected cell counts were <5.

a PQ-16 total score < 6 (latest wave).

b PQ-16 total score ≥ 6 (latest wave).

c Test statistic represents *t* values or Mann–Whitney *U* statistics for continuous variables and χ^2^ values for categorical variables.

d The denominator is the full sample per group; participants who did not report taking any medication were coded as non-users for each class. Medication classes: *antipsychotics* = risperidone, pipamperone, aripiprazole, pimozide, quetiapine, olanzapine, haloperidol; *antidepressants* = fluvoxamine, citalopram, paroxetine, fluoxetine, venlafaxine, bupropion, sertraline, mirtazapine, escitalopram, amitriptyline, nortriptyline, duloxetine, clomipramine; *benzodiazepines* = oxazepam, lorazepam, diazepam, clonazepam, temazepam, alprazolam; *stimulants* = methylphenidate, dexamphetamine; *Z-drugs* = zolpidem; *mood stabilizers/AEDs* = valproate; *non-stimulant ADHD meds* = atomoxetine, clonidine.

e Fisher’s exact test was used due to expected cell counts <5.

### Item-Level Patterns of PQ-16 Endorsement

To better understand the nature of these elevated symptom profiles, we next examined item-level endorsement patterns. The most commonly endorsed items included heightened sensory sensitivity (“suddenly distracted by distant sounds,” 50.9%) and intense internal experiences (“my thoughts are sometimes so strong that I can almost hear them,” 44.8%). In contrast, more overt psychosis-like experiences, such as “seeing faces change” (6.1%) or “seeing special meanings in objects” (14.5%), were relatively rare. Endorsement patterns varied substantially by group: For instance, among participants in the high symptom severity group (PQ-16 ≥ 6), 82.5% endorsed being distracted by distant sounds and 74.8% endorsed intense thoughts, compared to just 13.6% and 11.3% in the low symptom severity group. Full item-level frequencies and group comparisons are provided in Supplementary Table S1 and Figure S1.

### Predictors of PQ-16 Symptom Severity

To examine predictors of PQ-16 symptom severity, 3 linear regression models were estimated. In Model 1, younger age (β = −.243, *P* < .001) and higher autistic trait severity (AQ-28 total; β = .179, *P* < .001) were significantly associated with higher PQ-16 scores, whereas sex at birth (*P* = .076) and age at autism diagnosis (*P* = .104) were not significant predictors. Model 2 showed that specific trait domains, particularly Numbers & Patterns (β = .196, *P* < .001), Routine (β = .180, *P* = .001), Social Skills (β = .185, *P* = .002), and Switching (β = .184, *P* < .001), were uniquely associated with higher PQ-16 symptom severity. In the final model (Model 3; *n* = 867), depressive symptoms (PHQ-Dep-4; β = .321, *P* < .001) and autistic traits (AQ-28 total; β = .111, *P* < .001) remained significant independent predictors. Age, sex at birth, age at diagnosis, and employment status were not significant. The final model explained 13.2% of the variance (adjusted *R*^2^ = .132). See Table 3 for detailed results.

**Table 3:** Linear Regression Models Predicting Prodromal Psychotic Symptoms (PQ-16 Total Score).

**Model**	**Predictor**	***B***	**SE *B***	**β**	***t***	***P***	**95% CI LL**	**95% CI UL**	***R*** ^**2**^	**Adj. *R*** ^**2**^	***F*(df1, df2)**
**1**	Intercept	4.974	0.300	—	16.602	<.001	4.387	5.562	.050	.047	20.31 (4, 1554)^***^
	Z Age (latest)	–0.796	0.182	–0.243	–4.368	<.001	–1.154	–0.439			
	Sex (birth)	–0.313	0.177	–0.047	–1.775	.076	–0.660	0.033			
	Z Age at Diagnosis	0.287	0.177	0.088	1.626	.104	–0.059	0.634			
	Z AQ-28 Total	0.589	0.083	0.179	7.122	<.001	0.427	0.752			
**2**	Intercept	4.738	.298	—	15.919	<.001	4.154	5.321	.096	.091	18.29 (9, 1549)^***^
	Z Age (latest)	−0.609	0.181	−0.186	−3.370	<.001	−0.964	−0.255			
	Sex (birth)	−0.167	0.176	−0.025	−0.949	.343	−0.511	0.178			
	Z Age at Diagnosis	0.128	0.175	0.039	0.733	.464	−0.215	0.471			
	Z AQ–28: Numbers & Patterns	0.642	0.083	0.196	7.754	<.001	0.479	0.805			
	Z AQ–28: Routine	0.590	0.181	0.180	3.259	.001	0.235	0.945			
	Z AQ–28: Switching	0.601	0.175	0.184	3.427	<.001	0.257	0.601			
	Z AQ–28: Social Skills	0.605	0.194	0.185	3.119	.002	0.224	0.605			
	Z AQ–28: Imagination	0.269	0.278	0.083	0.971	.332	–0.276	0.814			
	Z AQ–28: Social Behavior	–1.223	0.538	–0.371	–2.274	.023	–2.279	–0.167			
**3**	Intercept	4.209	0.511	—	8.236	<.001	3.207	5.211	.139	.132	19.82 (7, 859)^***^
	Z Age (latest)	−0.039	0.254	−0.012	−0.152	.879	−0.537	0.459			
	Sex (birth)	−0.293	0.221	−0.045	−1.328	.185	−0.726	0.139			
	Z Age at Diagnosis	−0.151	0.240	−0.047	−0.628	.530	−0.622	0.320			
	Z AQ-28 Total	0.357	0.107	0.111	3.336	<.001	0.147	0.567			
	Z PHQ-Dep-4 Total	1.032	0.110	0.321	9.371	<.001	0.816	1.248			
	Employment status	0.078	0.211	0.012	0.367	.714	−0.337	0.493			

Models estimated via OLS with listwise deletion. Continuous predictors were z-standardized. Categorical variables: sex at birth (1 = male, 2 = female/other), and employment (1 = yes, 2 = no).VIFs <6 for all models. Model 1 = demographics + AQ-28 total; Model 2 = demographics + AQ-28 subscales; Model 3 = Model 1 + PHQ-Dep-4 + functioning.

^*^ *P* < .05, ^**^P < .01, ^***^P < .001.

### Psychosis Onset Predictors

Among participants without psychosis at baseline (wave 8; *n* = 361), 1.4% (*n* = 5) developed a psychosis-spectrum disorder at wave 9. All new-onset cases occurred only among individuals scoring above the PQ-16 cutoff at wave 8, with an incidence of 3.2% in the high-symptom severity group versus 0.0% among those below the cutoff (Fisher’s exact test, *P* = .015). A logistic regression showed that PQ-16 total scores significantly predicted new-onset psychosis (*OR* = 1.44, 95% *CI* [1.13, 1.84], *P* = .003), though sensitivity was low due to the low base rate (1.4%, *n* = 5). The model had a good fit (Hosmer–Lemeshow *P* = .956) and explained 18.7% of the variance (Nagelkerke *R^2^* = .187). Additional models examining distress scores and combined symptom/distress predictors were preregistered and reported in Table 4. Still, they yielded comparable results with similarly limited sensitivity due to the small number of transitions. In age-restricted sensitivity analyses (≤40 years at baseline; *n* = 133), 2 participants (1.5%) developed an SSD at follow-up. Consistent with the main analysis, all incident cases occurred among individuals scoring above the PQ-16 cutoff at baseline (3.4% vs 0.0%). However, this association was not statistically significant (Fisher’s exact test, *P* = .188), reflecting the limited statistical power due to the small number of events.

**Table 4:** Summary of Logistic Regression Models Predicting New Psychosis Diagnosis at Wave 9.

**Model**	**Predictors**	**χ** ^**2**^ **(df)**	**Nagelkerke *R*** ^**2**^	**H–L Test χ** ^**2**^ **(df), *P***	**Accuracy**	**Sensitivity**	**Specificity**
**Model 1**	PQ-16 Total Score (Wave 8)	9.26 (1)^**^	.187	3.59 (8), *P* = .892	98.6%	0.0%	100%
**Model 2**	PQ-16 Distress Score (Wave 8)	6.67 (1)^*^	.131	5.96 (8), *P* = .652	98.8%	0.0%	100%
**Model 3**	PQ-16 Total + Distress Scores (Wave 8)	9.26 (2)^*^	.187	3.59 (8), *P* = .892	98.6%	0.0%	100%

H–L = Hosmer–Lemeshow goodness-of-fit test. Sensitivity = % of true psychosis cases correctly identified; Specificity = % of non-cases correctly classified.

*N* = 356 for Models 1 and 3; *N* = 409 for Model 2 (due to missing data on distress scores).

^*^ *P* < .05, ^**^P < .01.

### ROC Analyses

Receiver operating characteristic analyses suggested apparent discriminative ability of PQ-16 scores for predicting new-onset schizophrenia-spectrum diagnoses. The total symptom score yielded an *AUC* of 0.867 (95% *CI* [0.754, 0.981]), while the distress score achieved an *AUC* of 0.896 (95% *CI* [0.829, 0.963]). A combined model incorporating both total and distress scores produced a similar estimate (*AUC* = 0.856, 95% *CI* [0.729, 0.982]). However, these estimates should be interpreted cautiously, given the very small number of incident cases (*n* = 5), which may lead to unstable predictive estimates. All models showed low sensitivity in identifying actual positive cases, reflecting the low base rate of psychosis onset in this sample. Full metrics and cutoffs are provided in Table 5 and ROC curves in Supplemetary Figure S2.

**Table 5:** Predictive Performance of PQ-16 Models for New-Onset Psychosis at Wave 9 Based on ROC Curve Analysis

**Model**	**Predictors included**	**AUC**	**Gini**	**Max Youden (K-S)**	**Optimal cutoff**
**1**	PQ-16 Total Score (Wave 8)	0.867	0.734	0.600 – 0.143 = 0.457	8.5
**2**	PQ-16 Distress Score (Wave 8)	0.896	0.792	0.800 – 0.177 = 0.623	11.5
**3**	PQ-16 Total + Distress (Combined)	0.856	0.712	1.000 – 0.297 = 0.703	0.012

Abbreviations: AUC = Area under the ROC curve; Gini = AUC × 2 – 1; Max Youden = Maximum Youden’s Index [sensitivity – (1 – specificity)]; Optimal cutoff = score or predicted probability that maximizes Youden’s Index. For Models 1 and 2, cutoffs refer to the raw PQ-16 scores; for Model 3, they refer to the predicted probability from the combined model.

### Exploratory Analysis

We conducted exploratory analyses to examine whether schizophrenia-spectrum outcomes differed by age of autism diagnosis, using a cutoff of 12 years to distinguish childhood-diagnosed (*n* = 200; 11.9%) from adulthood-diagnosed participants (*n* = 1481; 88.1%). Among participants without psychosis at baseline (wave 8; *n* = 361), new-onset psychosis between wave 8 and wave 9 was observed in 1.6% (5/319) of adulthood-diagnosed participants and in 0% (0/42) of childhood-diagnosed participants. However, this difference was not statistically significant (Fisher’s exact = 1.000, *P* = .414). Rates of elevated attenuated psychotic symptoms (PQ-16 ≥ 6) also showed no significant difference by diagnostic timing: 38.0% (76/200) of childhood-diagnosed and 33.6% (497/1481) of adulthood-diagnosed participants scored above the cutoff (χ^2^(1) = 1.55, *P* = .214).

An additional exploratory sensitivity analysis was conducted to examine whether antipsychotic use influenced PQ-16 symptom levels. Participants using antipsychotics (*n* = 198) reported modestly higher PQ-16 total scores than non-users (M = 5.08, SD = 3.41 vs M = 4.41, SD = 3.23; *t*(1679) = −2.73, *P* = .006, Cohen’s *d* = 0.21).

## Discussion

Similar to other autistic cohorts, in this large national sample of autistic adults (*N* = 1686), attenuated psychotic symptoms as measured by the PQ-16 were common, with approximately one-third of participants (34.1%) exceeding the validated cutoff (≥6). Elevated scores were associated with younger age and greater autistic trait severity, particularly in domains related to cognitive rigidity (eg, Numbers & Patterns, Switching). These associations were held in both stratified comparisons and multivariate regression models. Individuals above the cutoff were also more likely to identify outside the binary gender categories and to be unemployed, suggesting that prodromal symptom burden may co-occur with broader psychosocial vulnerability.

Item-level analyses further revealed substantial variability in PQ-16 symptom endorsement. The most frequently endorsed items reflected heightened sensory sensitivity and vivid internal experiences, such as being distracted by distant sounds (50.9%) or having thoughts that felt almost audible (44.8%). In contrast, classically psychotic-like experiences, including visual distortions or delusional beliefs, were relatively rare. This heterogeneity in item endorsement highlights that elevated PQ-16 scores in autistic adults are not uniformly driven by overt psychosis-like phenomena.

In longitudinal analyses, PQ-16 total scores significantly predicted new-onset schizophrenia-spectrum diagnoses over 1 year of follow-up. All incident cases (1.4% of the longitudinal subsample) occurred among individuals who had initially scored above the PQ-16 cutoff, yielding transition rates of 3.2% in the high-symptom severity group and 0.0% in the low-symptom severity group. Logistic regression confirmed a significant predictive effect (OR = 1.44), and ROC analyses showed apparent discriminative performance, particularly for distress scores. However, due to the low number of observed transitions (*n* = 5), these preliminary estimates may be unstable and prone to inflation in rare-event prediction contexts, and require replication in independent samples before informing clinical decision-making. Age-restricted sensitivity analyses yielded similar results, with all incident cases occurring in the higher symptom group, although the difference did not reach statistical significance due to the small number of events.

The prevalence of elevated PQ-16 scores observed in this cohort (34.1%) is substantially higher than rates reported in non-autistic populations. For comparison, community studies typically report PQ-16 positivity in approximately 20% of adolescents at self-reported risk, and meta-analytic estimates suggest that fewer than 2% of the general population meet clinical high-risk criteria based on structured interviews.^39^^,^^40^ This discrepancy aligns with prior work indicating that subclinical psychotic-like experiences are relatively common in autistic individuals, although findings across studies have been heterogeneous. Some investigations report elevated rates of perceptual anomalies and unusual beliefs in autism,^24^ whereas others find no group-level increase in positive symptoms, underscoring marked variability in symptom expression.^25^

Item-level findings from the present study help contextualize this heterogeneity observed across prior research. The predominance of endorsements related to sensory sensitivity and vivid internal experiences, paired with relatively low endorsement of overt psychotic-like phenomena, suggests that elevated PQ-16 scores in autistic adults may often reflect autism-related sensory and cognitive styles rather than an imminent prodromal psychosis state. This interpretation aligns with concerns raised in previous work that psychosis screening instruments may over-identify risk when applied to autistic populations without careful phenomenological differentiation. Examining symptom profiles at the item level, therefore, appears critical for distinguishing autism-related experiences from psychosis-relevant signals.

Regression analyses further clarified the clinical correlates of the attenuated psychotic symptoms. In multivariable models, greater overall autistic trait severity and higher levels of depressive symptoms emerged as independent predictors of PQ-16 scores. When autistic traits were examined at the domain level, dimensions related to cognitive rigidity, reduced flexibility, and systemizing tendencies showed strong univariate and domain-specific associations with the attenuated psychotic symptom severity. These findings align with prior literature highlighting shared neurocognitive and affective mechanisms across autism and SSDs, particularly in domains such as executive control, salience processing, and sensory integration.^8–10^^,^^17^

The prominent contribution of depressive symptoms, highly prevalent in autistic adults, supports the interpretation that psychotic-like experiences in autism may arise from broader affective–cognitive vulnerability rather than representing isolated markers of emerging psychosis.

These findings also raise important considerations regarding discriminant validity. The strong associations between PQ-16 scores and autistic trait dimensions, particularly cognitive rigidity and systemizing tendencies, suggest that the PQ-16 may partly capture autism-related cognitive styles or a broader transdiagnostic vulnerability profile rather than psychosis-specific risk alone. This interpretation is consistent with evidence that psychotic-like experiences are associated with multimorbid psychopathology and overall clinical severity. For example, individuals scoring above the PQ-16 cutoff have been shown to exhibit substantially higher levels of comorbid psychiatric disorders, indicating that PQ-16 scores may index general psychopathology burden rather than a narrowly defined prodromal psychosis state.^41^ Accordingly, elevated PQ-16 scores in autistic adults should be interpreted in a dimensional and contextual manner, rather than as isolated indicators of risk of imminent psychosis.

Although the PQ-16 demonstrated good psychometric performance, the findings highlight an important interpretative caveat. Several PQ-16 items capture experiences that may reflect autistic cognitive or sensory styles, raising the risk of false positives when the instrument is applied without contextual interpretation. The limited sensitivity for predicting diagnosed psychosis in this cohort underscores this concern. These results echo critiques suggesting that existing psychosis screening tools, developed largely in non-autistic or help-seeking samples, may overpathologize autism-related features when used without adaptation.^42^ Item-level reporting, as provided in the Supplementary Materials, offers 1 strategy for improving interpretability but does not fully resolve this limitation.

Clinically, the PQ-16 may still serve a useful role as a first-step screener in autistic adults, particularly for identifying individuals experiencing distressing perceptual or cognitive phenomena who may benefit from further assessment. However, elevated scores should not be interpreted as indicating risk of imminent psychosis in isolation. Instead, high PQ-16 scores in this population are more appropriately viewed as signals warranting careful, autism-informed clinical follow-up, ideally incorporating structured interviews and consideration of mood symptoms, sensory sensitivities, and developmental context.

From a research perspective, these findings underscore the need for psychosis screening approaches that are specifically calibrated for autistic populations. The strong associations between PQ-16 scores and autistic trait dimensions suggest that attenuated psychotic symptom measures may index a transdiagnostic vulnerability profile rather than a narrowly defined prodromal state in autism. Dimensional approaches that move beyond binary cutoffs and incorporate symptom distress, developmental baselines, and contextual interpretation may improve specificity and reduce false positives. Emerging autism-adapted instruments represent promising steps in this direction, but further validation is needed.^43^

This study represents the most comprehensive evaluation to date of the PQ-16 in autistic adults, leveraging a large, community-based cohort with substantial age and gender diversity and longitudinal follow-up. The availability of validated measures of autistic traits, depressive symptoms, and functional outcomes enabled a multidimensional characterization of individuals with elevated prodromal symptoms. Replication of higher symptom burden among gender-diverse participants further supports calls for targeted research in this underserved group.

However, several limitations warrant consideration. The low incidence of new-onset psychosis (*n* = 5) limited statistical power for predictive analyses and may have produced unstable predictive estimates, with potential risk of model overfitting. Psychosis diagnoses were based on self-report rather than clinician-administered interviews, and no external validation of these reports is available in the NAR, potentially leading to diagnostic misclassification. The relatively high mean age of the sample limits generalizability to younger populations, which are typically represented in clinical high-risk research. The cohort also included a higher proportion of individuals assigned female at birth (63%) than typically reported in childhood autism cohorts, which may further limit generalizability to more male-dominated clinical samples. Additionally, most participants were diagnosed with autism in adulthood, constraining inferences about developmental timing. Next, a notable proportion of participants reported antipsychotic use, which is frequently prescribed for the management of behavioral and affective symptoms (eg, irritability, aggression, and emotional dysregulation), rather than for psychotic disorders per se.^44^ As such, their use in this cohort should not be interpreted as indicative of underlying psychosis. This interpretation is consistent with prior work in the NAR demonstrating that many autistic adults show sustained and heterogeneous patterns of intervention use, including substantial reliance on medication, often in the context of co-occurring psychiatric conditions rather than psychosis-specific treatment.^45^ However, medication effects may have influenced both symptom reporting and the observed rate of transition to SSDs, potentially contributing to the low incidence of new-onset cases. Future studies should consider incorporating medication use as a covariate in longitudinal models. Finally, although the PQ-16 performed well psychometrically, its development in non-autistic samples may limit its clinical validity in autistic adults without targeted adaptation. Future studies should examine whether alternative age thresholds (eg, diagnosis before versus after age 16) or age-at-assessment stratification better capture variation in psychosis risk, particularly within the typical high-risk developmental window.

In conclusion, attenuated psychotic symptoms are common among autistic adults and are meaningfully associated with autistic traits, depressive symptoms, and functional outcomes. The PQ-16 captures these patterns effectively but shows limited sensitivity for predicting psychosis onset in low-base-rate populations. These findings highlight both the value and the limitations of brief self-report screeners in autism and underscore the need for refined, autism-informed approaches to psychosis risk assessment.

## Supplementary Material

Web_Material_sbag160

## Data Availability

Supplementary materials related to this article, including additional descriptive statistics, alternative model outputs, and code documentation, are available upon request. Requests should be directed to the corresponding author at d.basic@vu.nl.
