# Supplementary material for: Evaluating the Prodromal Questionnaire-16 for Screening and Prediction of Psychosis in Autistic Adults: Cross-Sectional and Longitudinal Findings From a National Cohort
Source: Schizophr Bull. 2026 Sep 16;52(5):sbag160. doi: 10.1093/schbul/sbag160 (PMC13586408; doi:10.1093/schbul/sbag160)
Supplement: Supplementary file 1 [file web_material_sbag160.zip › 20260713_PQ16_Autism_Schizophrenia_Bulletin__Supplementary_Materials_sbag160.docx]

**Supplementary Materials**

**Supplementary Table S1.** Endorsement Rates of Individual PQ-16 Items Among Autistic Adults (2023 Data, N = 1,686)

| **PQ-16 Item** | **Total N (%)** | **Low Risk**  **N (%)** | **Medium Risk N (%)** | **High Risk**  **N (%)** |
| --- | --- | --- | --- | --- |
| 1. I feel uninterested in the things I used to enjoy. | 477 (36.1%) | 82 (19.3%) | 162 (35.5%) | 233 (53.1%) |
| 2. I often seem to live through events exactly as they happened before (déjà vu). | 392 (29.6%) | 23 (5.4%) | 116 (25.3%) | 253 (57.5%) |
| 3. I sometimes smell or taste things that other people can’t smell or taste. | 601 (45.5%) | 59 (13.9%) | 223 (48.7%) | 319 (72.5%) |
| 4. I often hear unusual sounds like banging, clicking, hissing, clapping or ringing in my ears. | 492 (37.2%) | 30 (7.1%) | 167 (36.5%) | 295 (67.0%) |
| 5. I have been confused at times whether something I experienced was real or imaginary. | 339 (25.6%) | 17 (4.0%) | 97 (21.2%) | 225 (51.1%) |
| 6. When I look at a person, or look at myself in a mirror, I have seen the face change right before my eyes. | 81 (6.1%) | 2 (0.5%) | 8 (1.8%) | 71 (16.2%) |
| 7. I get extremely anxious when meeting people for the first time. | 471 (35.7%) | 35 (8.3%) | 163 (35.7%) | 273 (62.0%) |
| 8. I have seen things that other people apparently can't see. | 240 (18.2%) | 4 (0.9%) | 53 (11.6%) | 183 (41.6%) |
| 9. My thoughts are sometimes so strong that I can almost hear them. | 593 (44.8%) | 48 (11.3%) | 215 (46.9%) | 330 (74.8%) |
| 10. I sometimes see special meanings in advertisements, shop windows, or in the way things are arranged around me. | 192 (14.5%) | 3 (0.7%) | 43 (9.4%) | 146 (33.1%) |
| 11. Sometimes I have felt that I’m not in control of my own ideas or thoughts. | 459 (34.7%) | 38 (8.9%) | 137 (30.0%) | 284 (64.4%) |
| 12. Sometimes I feel suddenly distracted by distant sounds that I am not normally aware of. | 674 (50.9%) | 58 (13.6%) | 252 (55.0%) | 364 (82.5%) |
| 13. I have heard things other people can't hear like voices of people whispering or talking. | 211 (15.9%) | 5 (1.2%) | 37 (8.1%) | 169 (38.3%) |
| 14. I often feel that others have it in for me. | 236 (17.8%) | 4 (0.9%) | 59 (12.9%) | 173 (39.2%) |
| 15. I have had the sense that some person or force is around me, even though I could not see anyone. | 199 (15.0%) | 8 (1.9%) | 45 (9.8%) | 146 (33.1%) |
| 16. I feel that parts of my body have changed in some way, or that parts of my body are working differently than before. | 181 (13.7%) | 4 (0.9%) | 42 (9.2%) | 135 (30.6%) |

***Note.*** Percentages indicate the proportion of participants within each symptom severity group who endorsed each PQ-16 item at the 2023 timepoint 9 (Wave 9). Severity groups were defined as follows: **Low severity** (0–2 symptoms), **Medium severity** (3–5 symptoms), and **High severity** (≥6 symptoms). Item endorsement was coded as binary (1 = symptom present). Missing values were excluded pairwise.

**Supplementary Figure S1.** Prevalence of PQ-16 Item Endorsements by Symptom Severity Group (Wave 9)

***Note.*** Bar graph showing the percentage of participants endorsing each of the 16 PQ-16 items, stratified by symptom severity group based on Wave 9 total scores: Low-severity (0–2 symptoms), Medium- severity (3–5 symptoms), and High- severity (≥6 symptoms). Each bar represents the proportion of individuals within that group who endorsed the item as “True”.

**Supplementary Figure S2.** Receiver Operating Characteristic (ROC) Curves for PQ-16 Models Predicting New-Onset Psychosis at Follow-Up

|   **A. PQ-16 Total Score (Model 1)** |   **B. PQ-16 Distress Score (Model 2)** |
| --- | --- |
|   **C. Combined PQ-16 Total + Distress Scores (Model 3)** |   **D. All ROC Curves Overlaid for Direct Comparison (Models 1–3)** |

***Note.*** ROC curves display the predictive performance of PQ-16 symptom total scores (Model 1), distress scores (Model 2), and combined symptom + distress scores (Model 3) for new-onset psychosis at follow-up (T9). Despite the low base rate (1.4%), all models showed strong discriminative ability. Optimal cutoffs were determined using Youden’s Index.

AUC values: Model 1 (Total score): 0.867, 95% CI [0.754, 0.981]; Model 2 (Distress score): 0.896, 95% CI [0.829, 0.963]; Model 3 (Combined): 0.856, 95% CI [0.729, 0.982].
